# Meta-Analysis of Hematological Biomarkers as Reliable Indicators of Soft Tissue Sarcoma Prognosis

**DOI:** 10.3389/fonc.2020.00030

**Published:** 2020-01-30

**Authors:** Long-Qing Li, Zhen-Hua Bai, Liang-Hao Zhang, Yan Zhang, Xin-Chang Lu, Yi Zhang, Yong-Kui Liu, Jia Wen, Jia-Zhen Li

**Affiliations:** ^1^Department of Orthopedic Surgery, The First Affiliated Hospital of Zhengzhou University, Zhengzhou, China; ^2^Department of Urology, The First Affiliated Hospital of Zhengzhou University, Zhengzhou, China

**Keywords:** soft tissue sarcoma, meta-analysis, hematological markers, prognosis, biomarker, inflammation

## Abstract

**Background:** Several recent studies have reported the reliable prognostic effect of hematological biomarkers in various tumors. Yet, the prognostic value of these hematological markers in soft tissue sarcoma (STS) remains inconclusive. Thus, the aim of this meta-analysis was to check the effect of hematological markers on the prognosis of STS.

**Methods:** We systematically searched for relevant papers published before October 2019 in the PubMed and EMBASE databases. Overall survival (OS) and disease-specific survival (DSS) were the primary outcome, whereas disease-free survival was the secondary outcome. A thorough study of hazard ratios (HR) and 95% of confidence intervals (CIs) was done for determining the prognostic significance.

**Results:** We performed 23 studies that comprised of 4,480 patients with STS. The results revealed that higher neutrophil-to-lymphocyte ratio (NLR), C-reactive protein (CRP), and platelet-to-lymphocyte ratio (PLR) were associated with poor OS/DFS (HR = 2.08/1.72, for NLR; HR = 1.92/1.75, for CRP, and HR = 1.86/1.61, for PLR). In contrast, a low lymphocyte-to-monocyte ratio (LMR) was relate to worse OS/DFS (HR = 2.01/1.90, for LMR). Moreover, pooled analysis illustrated that elevated NLR and CRP represents poor DSS, with HRs of 1.46 and 2.06, respectively. In addition, combined analysis revealed that higher Glasgow prognostic score (GPS) was linked to an adverse OS/DSS (HR = 2.35/2.77).

**Conclusion:** Our meta-analysis suggested that hematological markers (NLR, CRP, PLR, LMR, and GPS) are one of the important prognostic indicators for patients affected by high-grade STS and patients with the STS being located in the extremity.

## Introduction

### Rationale

Soft tissue sarcoma (STS) is a relatively rare, heterogeneous tumor derived primarily from the mesodermal layer. Approximately 12,750 new cases and 5,270 deaths were reported in 2019 (1, 2). Several prognostic factors including tumor size, depth, histologic tumor grade, and patient age have proven effective in guiding the design of treatment regimens for STS (3). Nevertheless, mortality in patients with high-grade tumors is nearly 50%, primarily due to development of locally relapsed or metastatic tumors. Hence, more accurate predictive factors are required to allow for development of personalized treatment plans for high risk patients (4). Identifying accurate and novel biomarkers will provide improved treatment options and surveillance methods for STS.

For these novel biomarkers to provide more accurate diagnosis of patients with high risk of recurrence and metastasis, they must be readily accessible via non-invasive procedures and cost-effective. Accumulating evidence suggests that inflammatory cells and proteins play a key role in tumor development (5). Inflammation in the tumor microenvironment promotes angiogenesis, tumor invasion, and metastasis, subverts both the adaptive and innate immune responses while also increasing tumor cell proliferation and enhanced survival (5, 6). Fortunately, clinical routine tests, many of which are readily available and consist of inexpensive hematological markers, such as the NLR, CRP, PLR, LMR, and Glasgow prognostic score (GPS), can reflect the systemic inflammatory status. Notably, the aforementioned markers show reliable prognostic value for various tumors (7–13).

### Objectives and Research Question

Inflammatory hematological biomarkers that have proven effective as prognostic factors in other tumors, may offer similar prognostic roles for STS. Although, several recent retrospective studies have demonstrated prognostic significance for some of these biomarkers in STS patients, the prognostic efficacy of several other markers have yet to be fully characterized. Therefore, the primary purpose of this meta-analysis was to explore the prognostic role of hematological biomarkers in STS.

## Methods

### Search Strategies

Published reports before October 2019 and available in PubMed and EMBASE were retrieved through a systematic literature search. The keywords were as follows: hematologic markers, neutrophil-to-lymphocyte ratio (NLR), C-reactive protein (CRP), platelet-to-lymphocyte ratio (PLR), lymphocyte-to-monocyte ratio (LMR), GPS, STS, prognosis, survival, and mortality. Since this is a meta-analysis and all data are collected from previously published studies, no ethical approval is required.

### Inclusion and Exclusion Criteria

The inclusion criteria were as follows: (1) diagnosis of STS based on pathological examination; (2) the study assessed the prognostic value for a minimum of one hematologic marker through overall survival (OS), disease-specific survival (DSS), and/or disease-free survival (DFS); (3) hazard ratio (HR) was employed with a 95% confidence interval (CI) to represent the prognostic value of biomarkers; (4) studies published in English.

Studies were excluded if: (1) reviews, letters, comments, and case reports; (2) subjects include patients with osteogenic tumors; (3) studies did not follow standard treatment guidelines (4) overlapping or duplicate studies; (5) studies not in English.

### Data Extraction and Quality Assessment

Two investigators (LL and ZB) independently selected these studies. Discrepancies were resolved by consensus, and the following information was extracted from each study: first author's name, publication year, country, number of patients, treatment method, tumor stage, cut-off value, and survival outcomes. HRs were primarily collected from multivariate analysis; in the case of no relevant data, univariate analysis was adopted. Two investigators used the Newcastle-Ottawa scale (NOS) to examine the quality of the reference articles. Studies with NOS scores ≥ 6 were included in our meta-analysis since they are considered as high-quality studies (14).

### Data Analysis

Considering the similar survival outcomes, we combined DSS, sarcoma-specific survival (SSS), cancer-specific survival (CSS), and regarded them as DSS. In addition, recurrence-free survival (RFS), progression-free survival (PFS), and DFS were combined as DFS. The hematological biomarkers-survival outcome relationship was assessed by means of studying hazard ratio and 95% CI. The Cochrane *Q-test* and *I*^2^ statistics were used to assess the heterogeneity among the studies. A random effects model (Der Simonian-Laird method) was employed in the case of any significant heterogeneity (*P* < 0.05 and *I*^2^ > 50%) (15), otherwise the fixed-effect model (Mantel-Haenszel method) was applied (16). In addition, subgroup analysis by treatment method, tumor stage, and ethnicity of NLR, CRP, and PLR was conducted. With the help of Stata software, version 12.0 (Stata corporation, College Station, TX, USA), publication bias was performed, whereas evaluation was completed by means of Begg's funnel plots, Egger's tests as well as the trim and fill method (17). Data analyses were conducted by RevMan5.3 (Cochrane Collaboration) and two-side *P* < 0.05 was considered to be statistically significant.

## Results

### Study Selection and Characteristics

**Figure 1:**
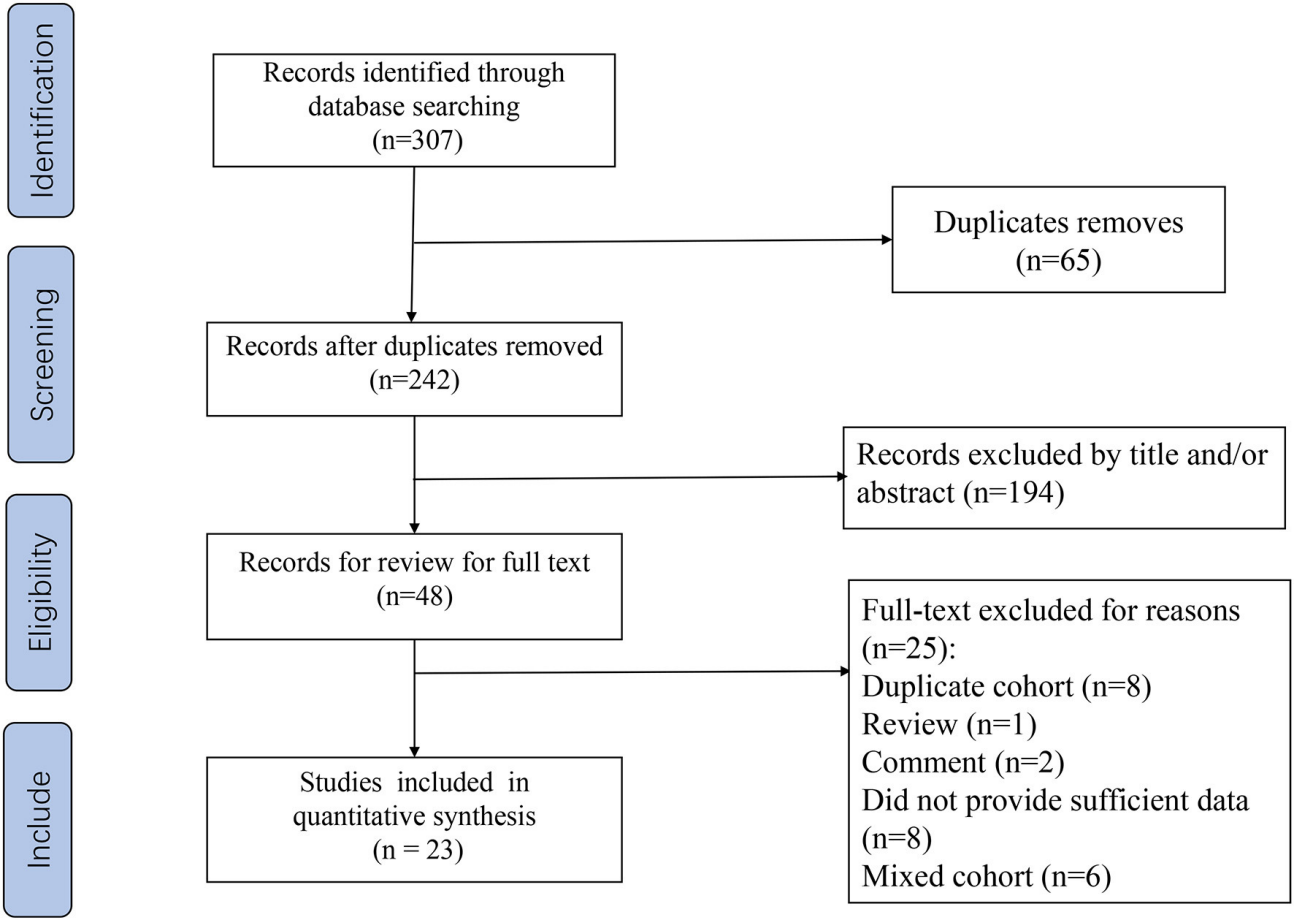
Flow chart of the included studies.

Our flow chart for data retrieval from publications is shown in Figure 1. The search strategy identified 307 potential records from the database. Ultimately, 23 studies involving 4,480 patients with STS met the inclusion criteria and were added into our meta-analysis. There were 15 studies for NLR, 11 for CRP, 7 for PLR, 4 for LMR, and 5 for GPS. The size of the samples ranged from 22 to 818. All studies collected data retrospectively. The mean NOS score was 6.95 and individual values ranged from 6 to 8. Further details of the studies are shown in Table 1.

**Table 1:** Baseline characteristics of studies included in the meta-analysis.

**References**	**Year**	**Country**	**Sample size**	**Treatment**	**Stage**	**Cut-off value**	**Makers**	**Outcome**
Idowu et al. (18)	2012	UK	83	Surgery	Non-metastatic	5	NLR	OS RFS
Marshall et al. (19)	2017	Japan	75	Mixed	Mixed	NA	CRP	OS
Nakamura et al. (20)	2012	UK	312	Surgery	Non-metastatic	10	CRP	DSS RFS
Szkandera et al. (21)	2014	Austria	170T/170V^*^	Surgery	Non-metastatic	5/200/2.85	NLR/PLR/LMR	OS DFS CSS
Panotopoulos et al. (22)	2015	Austria	85	Surgery	Mixed	NA/8.7	NLR/CRP	OS DSS
Jiang et al. (23)	2015	China	142	Mixed	Metastatic	1	NLR	OS PFS
Nakamura et al. (24)	2017	Japan	47	Mixed	Metastatic	5,3,2	CRP	DSS
Chan et al. (25)	2018	Singapore	529L/183M^†^	Surgery/Mixed	Non/Metastatic	2.5/184/2.4	NLR/PLR/LMR	OS RFS
Park et al. (26)	2019	Korea	99	Surgery	Non-metastatic	1.95/1.4	NLR/CRP	OS DFS
Sasaki et al. (27)	2018	Japan	103	Mixed	Mixed	5/NA/1	NLR/PLR/GPS	OS
Liang et al. (28)	2017	China	206	Surgery	Mixed	1.64/151.9/1	NLR/PLR/GPS	OS DFS
Maretty-Kongstad et al. (29)	2017	Denmark	818/403^‡^	Mixed	Non-metastatic	NA/NA/1	NLR/CRP/GPS	DSS
Nakamura et al. (30)	2015	Japan	139	Surgery	Non-metastatic	1	GPS	DSS EFS
Szkandera et al. (31)	2013	Austria	304	Surgery	Mixed	6.9	CRP	OS DFS CSS
Choi et al. (32)	2014	Korea	162	Surgery	Non-metastatic	2.5/2	NLR/CRP	DSS
García-Ortega et al. (33)	2017	Mexico	169	Mixed	Mixed	3.5	NLR	OS
Chen et al. (34)	2019	China	42	Surgery	Mixed	2.73/103.89/4.2	NLR/PLR/LMR	OS DFS
Willegger et al. (35)	2017	Austria	132	Surgery	Mixed	8.7	CRP	OS SSS RFS
Tsuda et al. (36)	2017	Japan	202	Surgery	Non-metastatic	1	GPS	SSS EFS
Vasquez et al. (37)	2017	Peru	22	Mixed	Mixed	2/150	NLR/PLR	OS
Nakamura et al. (38)	2017	Japan	81	Surgery	Mixed	2.8/14	NLR/CRP	DSS
Nakamura et al. (39)	2012	Japan	102	Mixed	Non-metastatic	3	CRP	DFS
Cheng et al. (40)	2019	China	103	Mixed	Mixed	2.7/154.99/4.16	NLR/PLR/LMR	OS/PFS

*NA, not available; OS, overall survival; DSS, disease-specific survival; SSS, sarcoma-specific survival; CSS, cancer-specific survival; DFS, disease-free survival; PFS, progression-free survival; RFS, recurrence-free survival*.

* *This study has validation set and training set, each set has 170 patients*.

† *This study has non-metastatic and metastatic group*.

‡ *Four hundred and three patients have data on CRP*.

### Synthesized Findings

#### Correlation Between NLR and OS/DSS/DFS in STS

**Figure 2:**
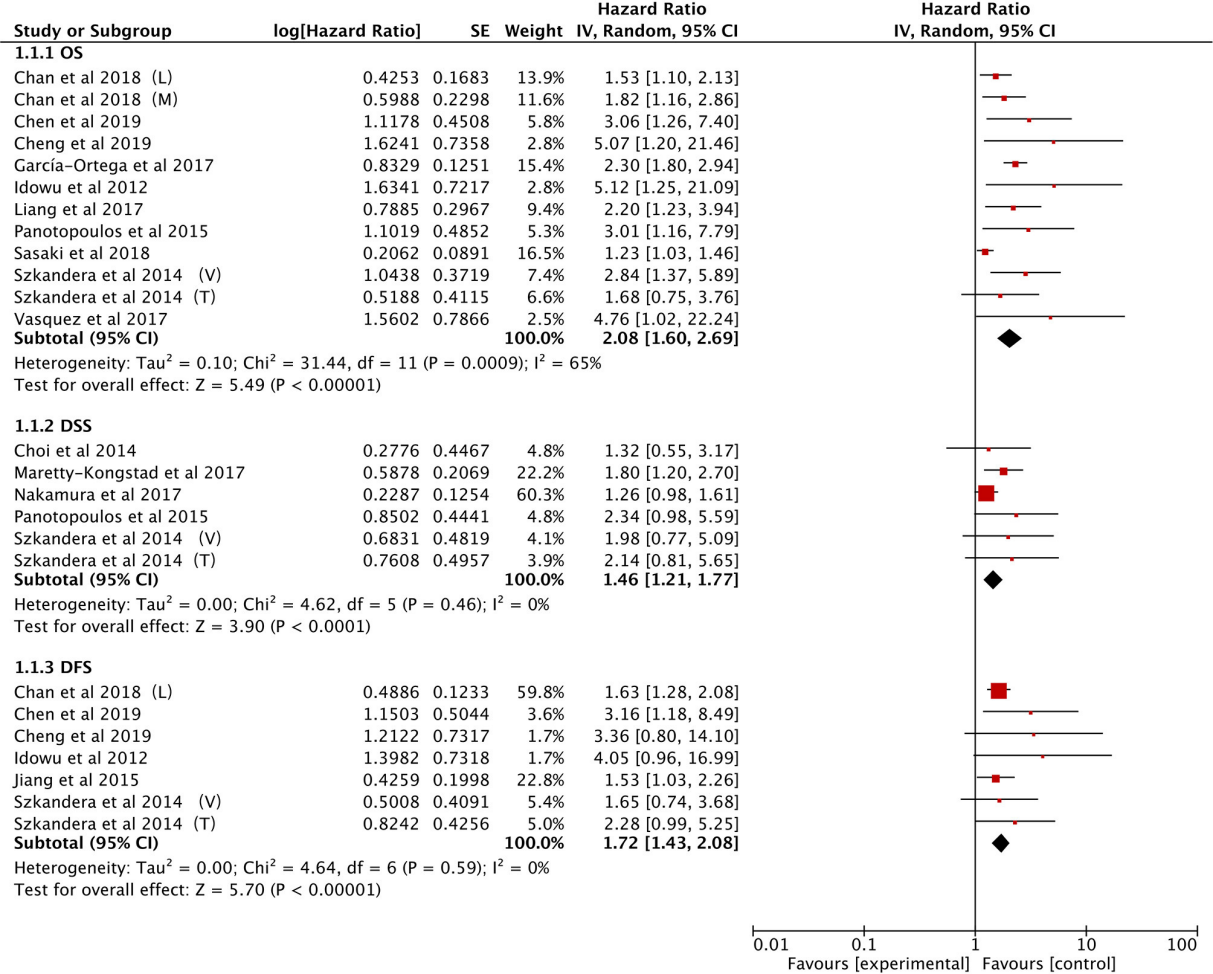
Forest plots of the Prognostic effect of NLR for OS/DSS/DFS.

**Figure 3:**
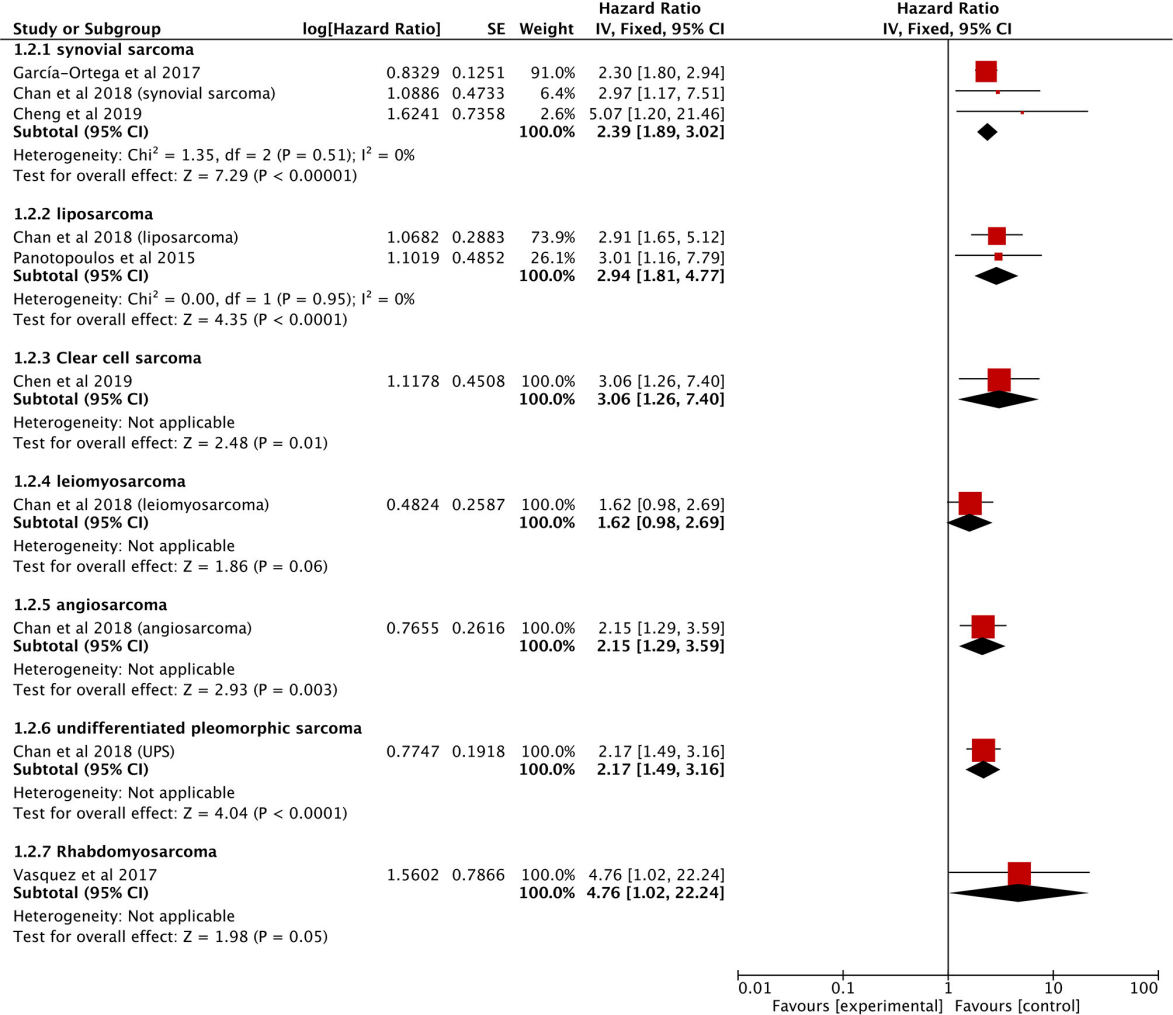
Forest plots of the Prognostic effect of NLR for OS in different histological subtypes.

The data on prognostic value of NLR for OS were reported in 10 studies holding 1,964 STS patients (18, 21, 22, 25, 27, 28, 33, 34, 37, 40). Overall, elevated NLR was significantly associated with poor OS (HR: 2.08, 95% CI: 1.60–2.69, *P* < 0.00001), and due to the moderate heterogeneity observed, a random effect model was used (*I*^2^ = 65%; Figure 2). The NLR-OS correlation in synovial sarcoma and liposarcoma was shown in three studies and two studies, respectively (HR: 2.39, 95% CI: 1.89–3.02, *P* < 0.00001 for synovial sarcoma; HR: 2.94, 95% CI: 1.81–4.77, *P* < 0.0001 for liposarcoma); no heterogeneity was detected (*I*^2^ = 0%; Figure 3). Only one study provided data on leiomyosarcoma, undifferentiated pleomorphic sarcoma, angiosarcoma, clear cell sarcoma, and rhabdomyosarcoma (HR: 1.62, 95% CI: 0.97–2.69, *P* = 0.087 for leiomyosarcoma; HR: 2.17, 95% CI: 1.49–3.16, *P* = 0.0002 for undifferentiated pleomorphic sarcoma; HR: 2.15, 95% CI: 1.29–3.59, *P* = 0.0056 for angiosarcoma; HR: 3.06, 95% CI: 1.26–7.40, *P* = 0.013 for clear cell sarcoma; HR: 4.76, 95% CI: 1.01–22.24, *P* = 0.024 for rhabdomyosarcoma).

The correlation between NLR and DSS was demonstrated in five studies comprising 1,486 STS patients (21, 22, 29, 32, 38). Collected data showed that poor prognosis of DSS was associated with high NLR (HR: 1.46, 95% CI: 1.21–1.77, *P* < 0.0001) without heterogeneity (*I*^2^ = 0%; Figure 2).

Six studies provided the data of NLR and DFS in STS (18, 21, 23, 25, 34, 40). The combined analysis indicated that NLR had a significant prognostic effect on DFS (HR: 1.72, 95% CI: 1.43–2.08, *P* < 0.00001), and no heterogeneity was detected (*I*^2^ = 0%; Figure 2).

Subgroup analysis illustrated that NLR was association with poor OS, DSS, and DFS in most subgroups, while the DSS Asia group had no significant prognostic value (Table 2).

**Table 2:** Subgroup analysis of the prognostic value of NLR.

**Survival analysis**	**No. of studies**	***I*^**2**^ (%)**	**HR (95% CI)**	***P***
**OS**				
Total	10	65%	2.08 (1.60–2.69)	*P* < 0.00001
**Treatment**				
Surgery	6^*^	14%	1.97 (1.56–2.48)	*P* < 0.00001
Mixed	5	82%	1.98 (1.27–3.08)	*P* = 0.002
**Stage**				
Non-metastatic	3^†^	33%	1.77 (1.34–2.33)	*P* < 0.0001
Metastatic	2	0%	2.06 (1.45–2.92)	*P* < 0.0001
Mixed	6	80%	2.33 (1.45–3.75)	*P* = 0.0005
**Ethnicity**				
Asian	5	59%	1.72 (1.29–2.31)	*P* = 0.0003
Latinos	2	0%	2.34 (1.84–2.98)	*P* < 0.00001
Caucasian	3	0%	2.60 (1.66–4.06)	*P* < 0.0001
**DSS**				
Total	5	0%	1.46 (1.21–1.77)	*P* < 0.0001
**Treatment**				
Surgery	4	0%	1.38 (1.11–1.71)	*P* = 0.004
Mixed	1	NA	1.80 (1.20–2.70)	*P* = 0.004
**Stage**				
Non-metastatic	3	0%	1.78 (1.29–2.46)	*P* = 0.0005
Mixed	2	45%	1.32 (1.04–1.67)	*P* = 0.02
**Ethnicity**				
Asian	2	0%	1.26 (1.00–1.60)	*P* = 0.05
Caucasian	3	0%	1.92 (1.39–2.66)	*P* < 0.0001
**DFS**				
Total	6	0%	1.72 (1.43–2.08)	*P* < 0.00001
**Treatment**				
Surgery	4	0%	1.76 (1.42–2.18)	*P* < 0.00001
Mixed	2	7%	1.62 (1.11–2.36)	*P* = 0.01
**Stage**				
Non-metastatic	3	0%	1.71 (1.37–2.13)	*P* < 0.00001
Metastatic	1	NA	1.53 (1.03–2.26)	*P* = 0.03
Mixed	2	0%	3.22 (1.43–7.27)	*P* = 0.005
**Ethnicity**				
Asian	4	0%	1.67 (1.37–2.04)	*P* < 0.00001
Caucasian	2	0%	2.14 (1.25–3.65)	*P* = 0.005

*NA, not available*.

* *Chan 2018's study has both surgery cohort and mixed treatment cohort*.

† *Chan 2018's study has both metastatic group and non-metastatic group*.

#### Prognostic Value of Elevated CRP for OS/DSS/DFS

**Figure 4:**
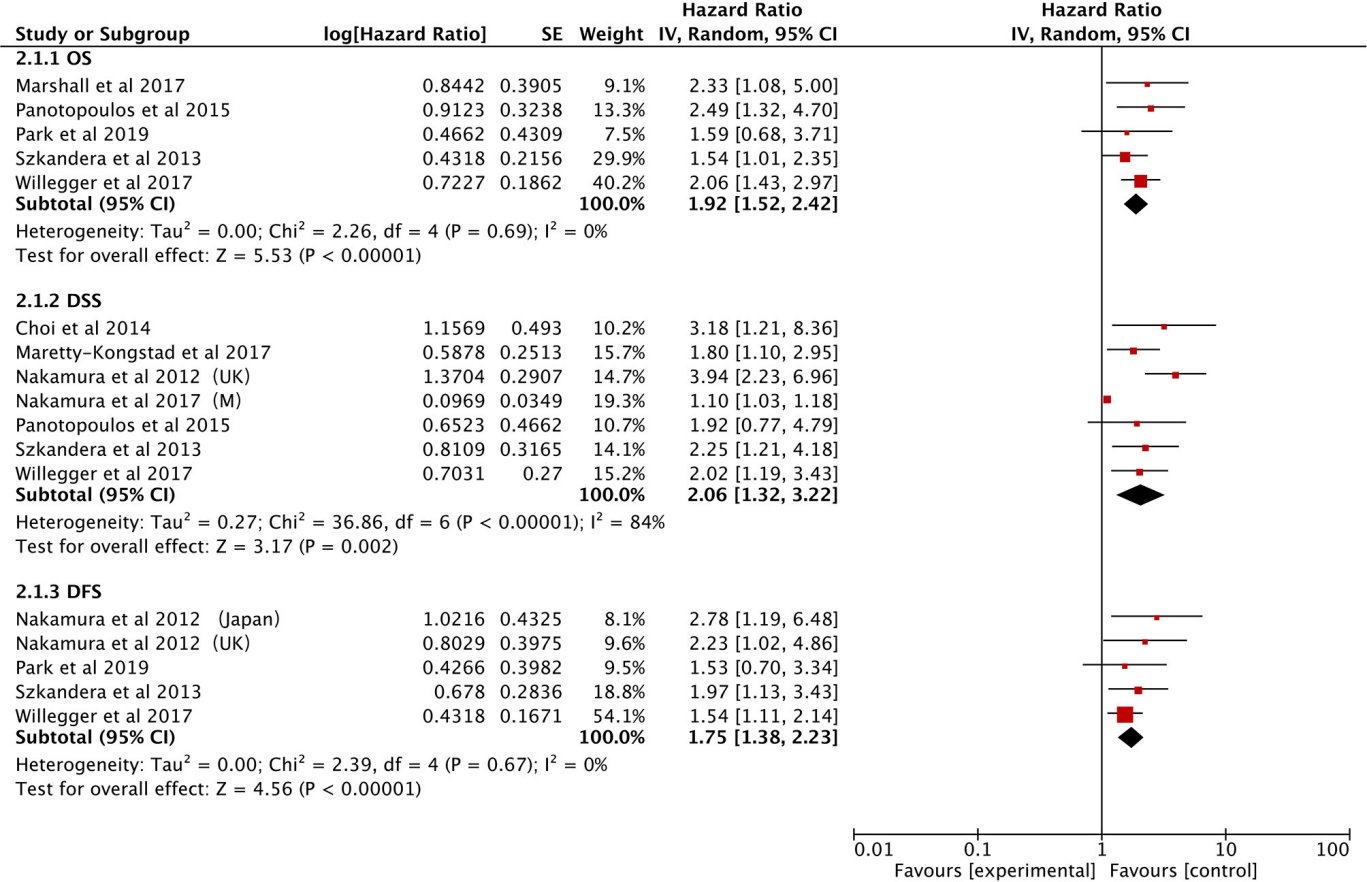
Forest plots of the Prognostic effect of CRP for OS/DSS/DFS.

The effect of CRP on the STS prognosis was demonstrated in five studies (19, 22, 26, 31, 35). The analysis showed that a higher CRP level is a useful prognostic marker for predicting survival rate (HR: 1.92, 95% CI: 1.52–2.42, *P* < 0.00001) with no heterogeneity between studies (*I*^2^ = 0%; Figure 4). Seven studies reported the data on CRP and DSS (20, 22, 24, 29, 31, 32, 35). The random-effects model demonstrated that an elevated CRP levels had significantly prognostic value for DSS (HR: 2.06; 95% CI: 1.32–3.22; *P* = 0.002), but with significant heterogeneity (*I*^2^ = 84.0%; Figure 4). The correlation between CRP and DFS was demonstrated in five studies, and the pooled data illustrated that an elevated CRP level was associated with poor DFS (HR: 1.75; 95% CI: 1.38–2.23; *P* < 0.00001) (20, 26, 31, 35, 39). No heterogeneity (*I*^2^ = 0%; Figure 4) was observed. Subgroup analysis is shown in Table 3. The non-metastatic group did not show significant significance with regard to OS; the mixed treatment group and Asian ethnicity group did not show significant significance with respect to DSS.

**Table 3:** Subgroup analysis of the prognostic value of elevated CRP.

**Survival analysis**	**No. of studies**	***I*^**2**^ (%)**	**HR (95% CI)**	***P***
**OS**				
Total	5	0%	1.92 (1.52–2.42)	*P* < 0.00001
**Treatment**				
Surgery	4	0%	1.88 (1.48–2.40)	*P* < 0.00001
Mixed	1	NA	2.33 (1.08–5.00)	*P* = 0.03
**Stage**				
Non-metastatic	1	NA	1.59 (0.68–3.71)	*P* = 0.28
Metastatic	4	0%	1.95 (1.53–2.48)	*P* < 0.00001
**Ethnicity**				
Asian	2	0%	1.96 (1.11–3.46)	*P* = 0.02
Caucasian	3	0%	1.91 (1.48–2.46)	*P* < 0.00001
**DSS**				
Total	7	84%	2.06 (1.32–3.22)	*P* = 0.001
**Treatment**				
Surgery	5	0%	2.57 (1.91–3.45)	*P* < 0.00001
Mixed	2	73%	1.32 (0.83–2.10)	*P* = 0.24
**Stage**				
Non-metastatic	3	54%	2.72 (1.57–4.69)	*P* = 0.0003
Metastatic	1	NA	1.10 (1.03–1.18)	*P* = 0.005
Mixed	3	0%	2.08 (1.44–3.01)	*P* < 0.0001
**Ethnicity**				
Asian	2	77%	1.68 (0.62–4.54)	*P* = 0.30
Caucasian	5	16%	2.29 (1.76–2.97)	*P* < 0.00001
**DFS**				
Total	5	0%	1.75 (1.38–2.23)	*P* < 0.00001
**Treatment**				
Surgery	4	0%	1.68 (1.31–2.16)	*P* < 0.0001
Mixed	1	NA	2.78 (1.19–6.48)	*P* = 0.02
**Stage**				
Non-metastatic	3	0%	2.09 (1.31–3.31)	*P* = 0.002
Mixed	2	0%	1.64 (1.24–2.18)	*P* = 0.0006
**Ethnicity**				
Asian	2	2%	2.01 (1.13–3.57)	*P* = 0.02
Caucasian	3	0%	1.70 (1.30–2.22)	*P* < 0.0001

*NA, not available*.

#### Prognostic Effect of PLR for OS/DFS

**Figure 5:**
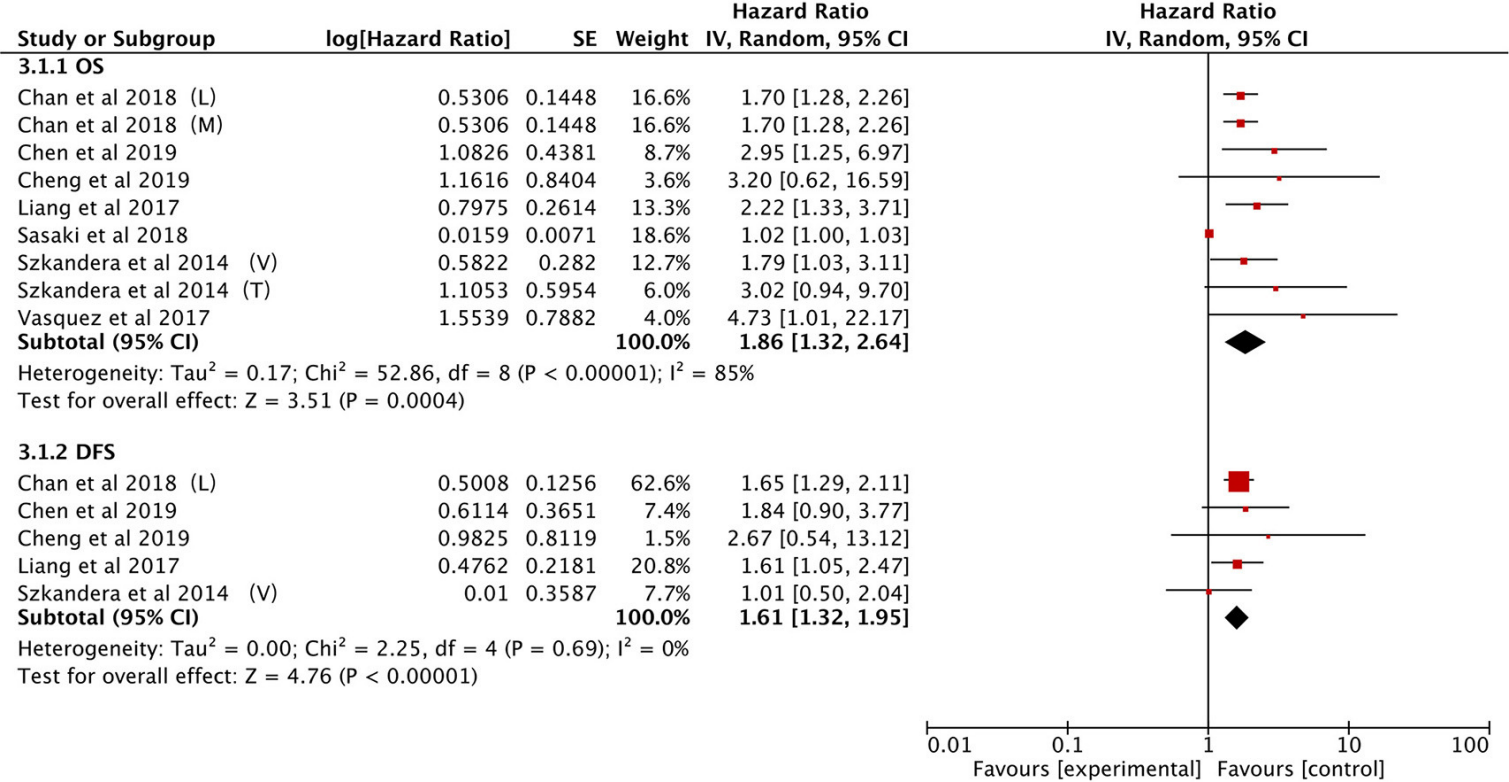
Forest plots of the Prognostic effect of PLR for OS/DFS.

The association between PLR and OS was demonstrated in seven studies (21, 25, 27, 28, 34, 37, 40). Elevated PLR was clearly associated with poor OS (HR: 1.86, 95% CI: 1.32–2.64, *P* = 0.0004), however, significant heterogeneity was observed (*I*^2^ = 85%; Figure 5).

The effect of PLR and DFS was reported in five studies (21, 25, 28, 34, 40). The fixed-effect model illustrated that an elevated PLR correlated with poor DFS (HR: 1.61, 95% CI: 1.32–1.95, *P* < 0.00001) with no heterogeneity among the studies (*I*^2^ = 0%; Figure 5).

Subgroup analytical studies illustrated that PLR had significant prognostic effect for OS and DFS in most subgroups, while the mixed treatment group on OS and DFS Caucasian ethnicity group had no significant prognostic value (Table 4).

**Table 4:** Subgroup analysis of the prognostic value of PLR.

**Survival analysis**	**No. of studies**	***I*^**2**^ (%)**	**HR (95% CI)**	***P***
**OS**				
Total	7	85%	1.86 (1.32–2.64)	*P* < 0.00001
**Treatment**				
Surgery	4	0%	1.90 (1.53–2.35)	*P* < 0.00001
Mixed	4	84%	1.55 (0.93–2.58)	*P* = 0.09
**Stage**				
Non-metastatic	2	0%	1.76 (1.38–2.26)	*P* < 0.00001
Metastatic	1	NA	1.70 (1.28–2.26)	*P* = 0.0002
Mixed	5	80%	2.09 (1.08–4.04)	*P* = 0.03
**Ethnicity**				
Asian	5	88%	1.72 (1.17–2.52)	*P* = 0.006
Caucasian	1	0%^*^	1.97 (1.20–3.25)	*P* = 0.008
Latinos	1	NA	4.73 (1.01–22.17)	*P* = 0.05
**DFS**				
Total	5	0%	1.61 (1.32–1.95)	*P* < 0.00001
**Stage**				
Non-metastatic	2	40%	1.56 (1.24–1.97)	*P* = 0.0002
Mixed	3	0%	1.71 (1.19–2.44)	*P* = 0.003
**Ethnicity**				
Asian	4	0%	1.67 (1.36–2.06)	*P* < 0.00001
Caucasian	1	NA	1.01 (0.50–2.04)	*P* = 0.98

*NA, not available*.

* *Szkandera 2014's study has validation set and training set, each set has 170 patients*.

#### Association Between LMR and OS/DFS in STS

**Figure 6:**
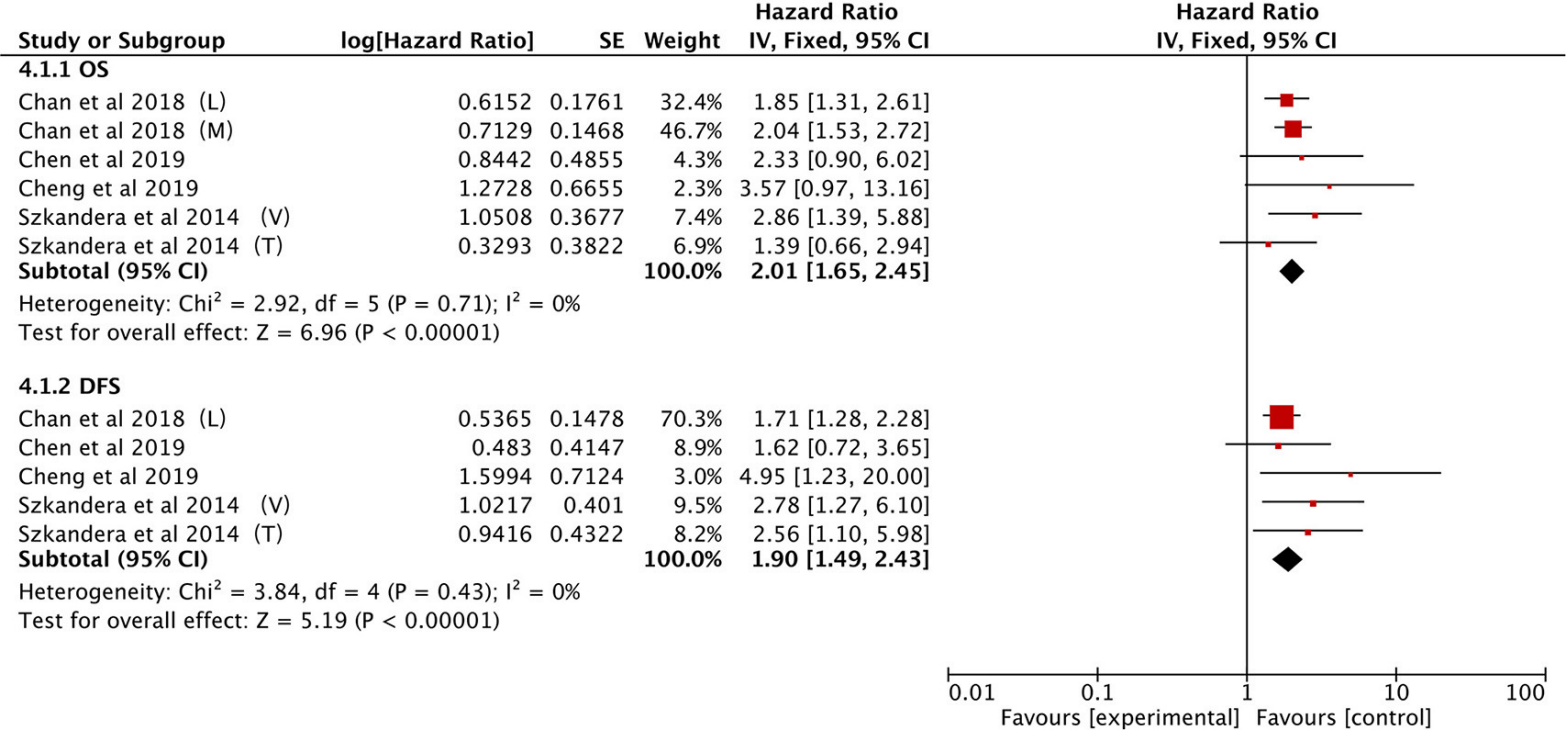
Forest plots of the Prognostic effect of LMR for OS/DFS.

A total of four studies provided LMR data on OS in STS patients (21, 25, 34, 40). The pooled data demonstrated that a low LMR had a visible prognostic effect on OS with an HR of 2.01 (95% CI: 1.65–2.45, *P* < 0.00001). No heterogeneity was observed (*I*^2^ = 0%; Figure 6).

The same four studies illustrated that LMR was also associated with DFS (21, 25, 34, 40). Alternatively, pooled data indicated that a low LMR had strong association with DFS (HR: 1.90, 95% CI: 1.49–2.43, *P* < 0.00001) and heterogeneity was not observed between studies (*I*^2^ = 0%; Figure 6).

#### Value of GPS for OS/DSS

**Figure 7:**
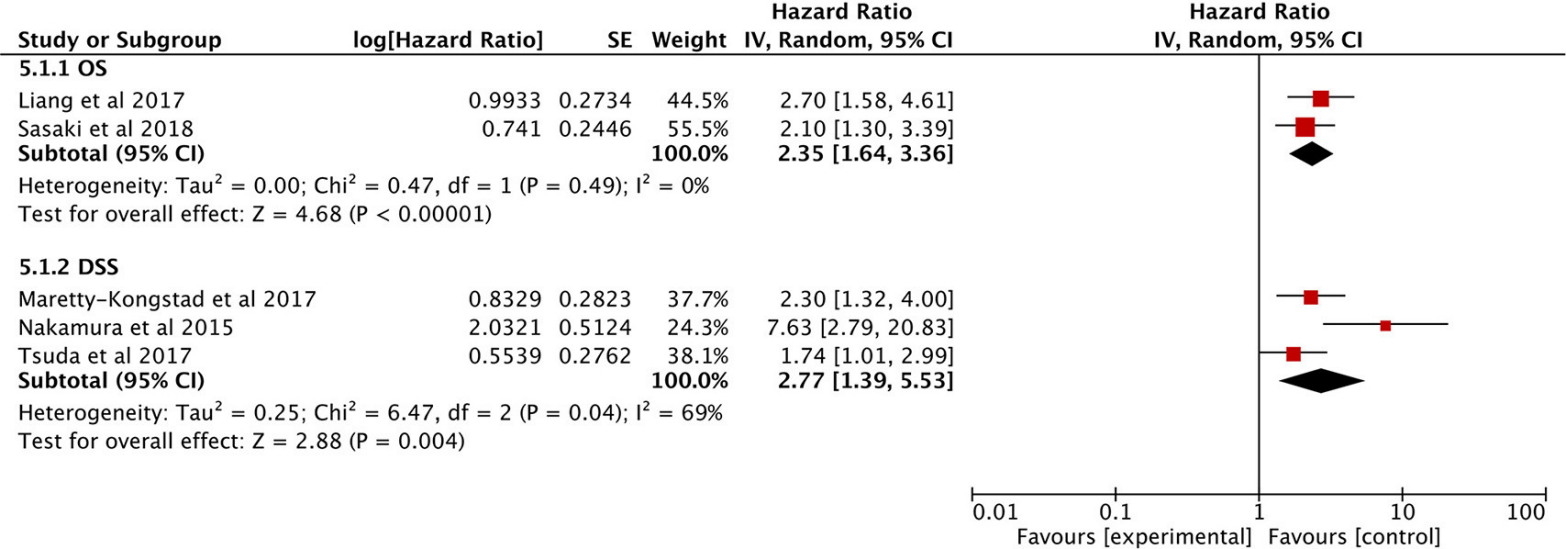
Forest plots of the Prognostic effect of GPS for OS/DSS.

Only two eligible studies explored the correlation between the GPS and OS (27, 28), and the combined data indicated that higher GPS scores correlated with much poorer OS (HR: 2.35; 95% CI: 1.64–3.36, *P* < 0.00001), without heterogeneity (*I*^2^ = 0%; Figure 7).

Three other studies show that high GPS is associated with poor DFS (29, 30, 36). The analysis showed that a higher GPS score is a useful prognostic marker for predicting DFS (HR: 2.77, 95% CI = 1.39–5.53, *P* = 0.004) with significant heterogeneity (*I*^2^ = 69%; Figure 7).

## Discussion

We performed a meta-analysis of 23 studies that were identified from multiple databases to examine the prognostic effect of hematological markers for STS. In our study, majority were high-grade and extremity tumors. The most common histological subtype was liposarcoma accounting for ~830 cases, followed by malignant fibrous histiocytoma/undifferentiated pleomorphic sarcoma with ~780 cases, and ~550 cases of synovial sarcoma. The pooled data indicated that hematological markers, comprising NLR, CRP, PLR, LMR, and GPS, were associated with survival outcomes of STS; while high NLR, CRP, PLR, and GPS as well as low LMR were correlated with poorer prognosis. The results of the subgroup analysis also support our conclusions. Yet, many of the patients in our study were high grade patients with tumors located in the extremities, hence, these results should not be applied to all patients with STS. Patients with non-extremity and low-grade tumors require further analysis. Collectively, our findings suggest that these established markers, which can be tested using inexpensive, readily available assays, may serve as important biomarkers for the prognosis of high grade and extremity STSs.

Recently, the treatment for STS has changed allowing for improved overall prognosis. Despite some limitations, the clinical and pathological features have served as the primary prognostic factors for STS in recent decades. Innovative methodology has to must be applied to achieve improved early diagnosis of patients at risk of a specific outcome with acceptable cost (41). Molecular markers have shown reliable prognostic value in numerous types of cancer, some of which, including MDM2, MMP2, and P53, also exhibit a certain prognostic value in STSs. The MDM2 gene has been widely used in the diagnosis of STSs. A number of clinical trials targeting MDM2 gene drugs have recently been conducted. Unfortunately, a meta-analysis shows that the MDM2 gene has a very limited role in prognosis (42, 43). Moreover, molecular detection technology must be improved to allow for reduced costs associated with evaluation (44, 45). Other markers, such as tumor necrosis, 18F-fluorodeoxyglucose positron emission tomography, and PD-1/PD-L1 have also demonstrated prognostic effects in STS. However, the clinical use of these markers is very limited (46, 47). Hence, none of these biomarkers are ready for clinical use.

In cancer patients, hematological markers serve as sensitive prognostic indicators, with inflammatory markers being the most reliable (7–13). The belief that a relationship exists between inflammation and tumor development can be traced back to the nineteenth century. As early as 1863, Rudolf Virchow observed leukocytes in tumor tissues and established this hypothesis. Due to the limitations of the times and technology, this speculation has been silent for many years. However, currently, our knowledge of inflammation in the tumor microenvironment has supported this hypothesis (48, 49). In fact, evidence now suggests that inflammation of the tumor microenvironment promotes tumorigenesis, growth, and metastasis, with a very prominent link between inflammation and tumors (5, 6, 49).

NLR is currently the most common hematological inflammation marker. Neutrophils can remodel the extracellular matrix and promote angiogenesis, which may stimulate tumor cell migration and metastasis. Furthermore, neutrophils significantly impact immunity by inhibiting cytolytic activity of lymphocytes, whereas tumor-infiltrating lymphocytes may restrict the metastatic outgrowth of cancer cells (50–52). In a previous study, Liu et al. (53) indicated that NLR may serve as a prognostic marker in both localized bone and STSs. However, osteoblastic tumors differ markedly from STSs in terms of treatment and prognosis. We, therefore, separated STS from osteogenic tumors and included a larger sample size.

The prognostic effect of CRP has been established in a variety of cancers. Tumor growth can lead to inflammation of tissues, thereby elevating the CRP level. Previous studies have preliminarily demonstrated the prognostic value of CRP in STS, however, there are certain limitations to these studies. For example, Li et al. (54) did not separate DSS from the OS even though these variable constitute two unique concepts by definition, especially when considering tumor prognosis. This can be observed from our conclusion. Compared to Xiaolin Wang's research (55), we have included more papers to provide a more comprehensive endpoint.

Previous studies have also shown that PLR exhibits reliable prognostic value in various tumors, such as those of ovarian cancer, pancreatic cancer, and bladder cancer. Platelets can mediate tumor cell growth, angiogenesis, and proliferation by releasing vascular endothelial growth factor, hepatocyte growth factor, basic fibroblast growth factor, angiopoietin-1 together with other angiogenesis and tumor growth factors. Furthermore, platelets have a defined role in protecting tumor cells from immune elimination and supporting tumor metastasis (56–58). In this meta-analysis, we observed that elevated PLR was clearly related with poor OS and DFS, consistent with the findings of previous studies. To our knowledge, this study is the first meta-analytical study that conducted research on the prognostic effect of PLR in STS patients.

Recent studies have also provided insights into the prognostic value of LMR. In fact, it has been suggested that LMR is a better prognostic indicator. Further, studies have highlighted the importance of tumor-associated macrophages. Hence, TMA derived from peripheral blood monocytes may support tumor progression and angiogenesis through secretion of growth factors and cytokines (59). This is also the first meta-analytical study, to our knowledge, to investigate LMR prognostic value in STS patients. However, only three studies were qualified for our analytical study, and subsequent studies are required.

There is also an increasing interest in scoring based on the inflammatory biomarkers. GPS is now used to predict various tumor prognoses (12). Glasgow's prognosis score consists of CRP and albumin as albumin levels in plasma reflect both the patient's nutritional level and systemic inflammation. However, most high scores are caused by abnormalities in CRP. Implying that the score is based on systemic inflammation. The significant correlation between GPS and STS is what our study demonstrated, with no similar meta-analysis previously performed.

Our study also has several limitations. First, we need to acknowledge that we cannot correct the histological subtype, a confounding factor that may affect outcomes. We have done our best to analyze histological subtypes. However, only three studies provided data on synovial sarcoma, two studies provided data on liposarcoma, and one provided data on clear cell sarcoma, angiosarcoma, undifferentiated pleomorphic sarcoma, rhabdomyosarcoma, and leiomyosarcoma. Results for a single subtype suggest that NLR has prognostic value in most subtypes, however, it is not possible to predict the prognosis of leiomyosarcoma. Thus, more research on specific subtypes is needed to further validate our results. Second, since some studies did not include multivariate analysis data, we included a portion of univariate analysis. Third, the same blood markers have different cut-off values. However, since there have been no studies to compare the prognostic effects of different cutoff values, the optimal value cannot be evaluated. Nevertheless, our meta-analysis is the largest study to investigate the prognostic value of hematological markers in STSs. Compared to previous studies, we have included a larger sample size and excluded confounding factor of osteogenic tumors. Moreover, we are the first, to our knowledge, to investigate the prognostic value of multiple markers in STSs. These factors reinforce the strengths of our meta-analysis.

## Publication BIAS

**Figure 8:**
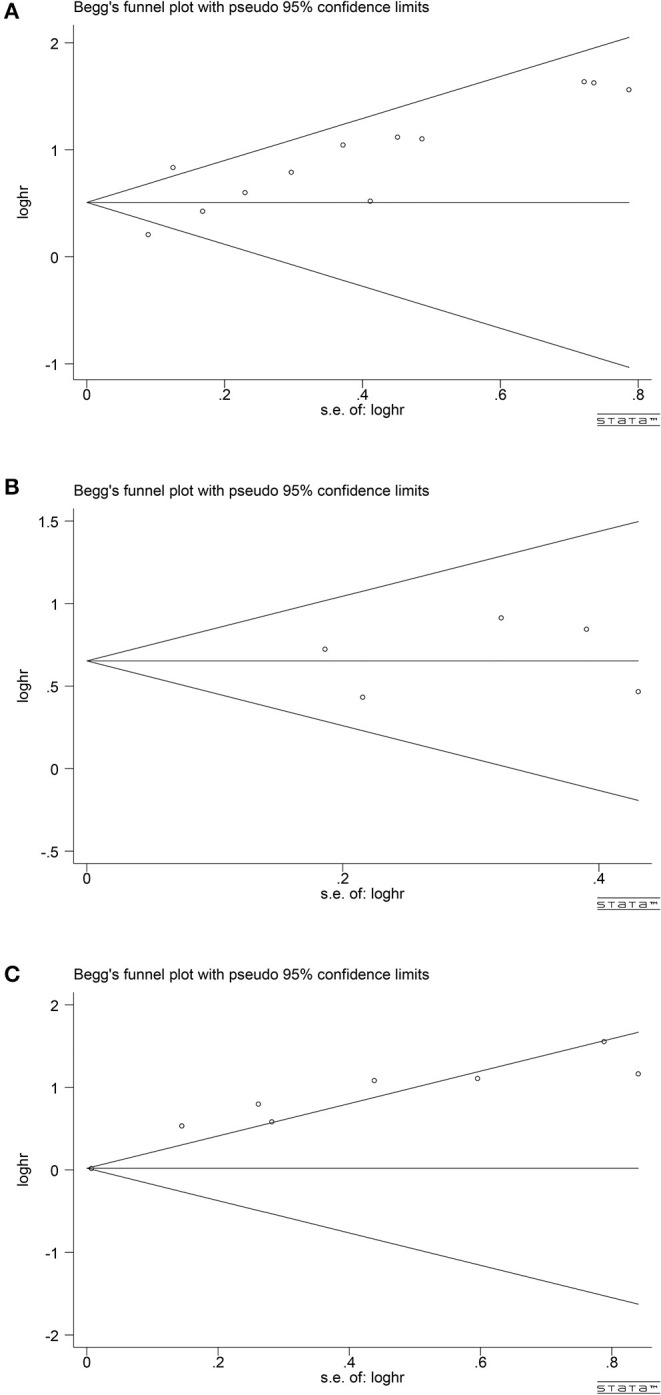
Analyses of publication bias for the relationship between NLR/CRP/PLR and OS **(A)** Begger's funnel plot for NLR. **(B)** Begger's funnel plot for CRP. **(C)** Begger's funnel plot for PLR.

**Figure 9:**
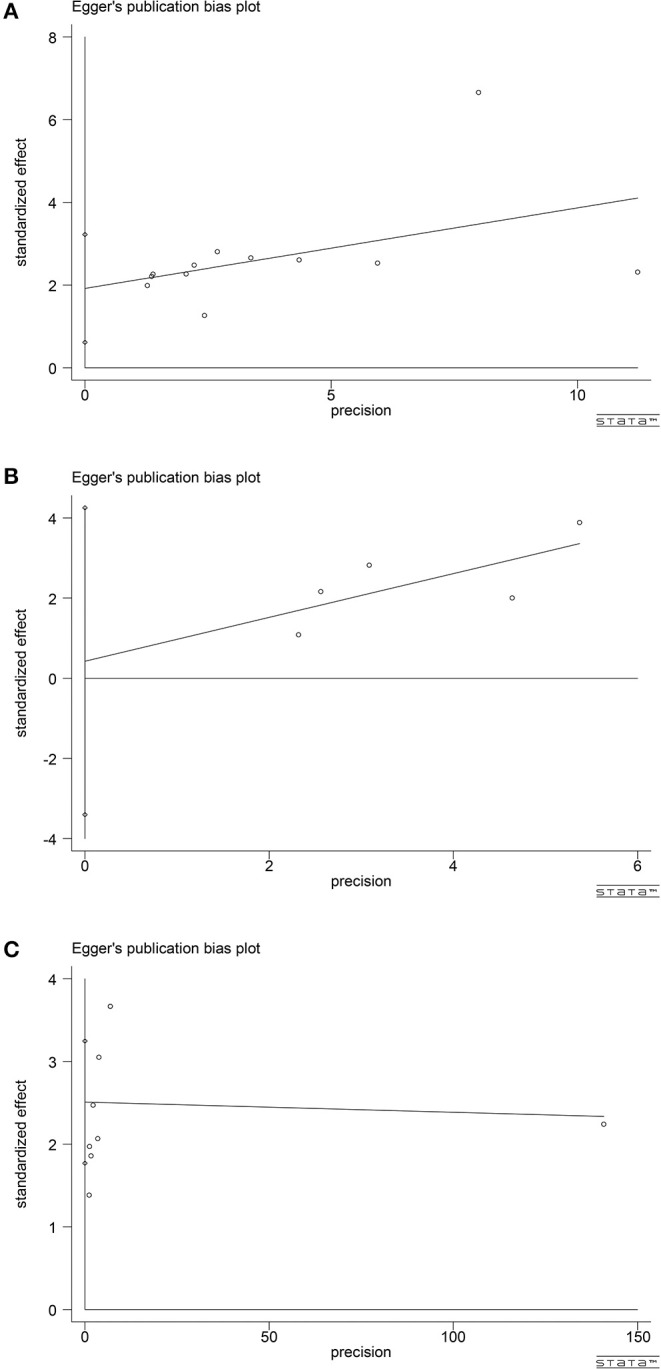
Analyses of publication bias for the relationship between NLR/CRP/PLR and OS **(A)** Egger's publication bias plot for NLR. **(B)** Egger's publication bias plot for CRP. **(C)** Egger's publication bias plot for PLR.

According to the publication-bias-plot shown in Figures 8, 9, the bias was insignificant with regards to the prognostic value of NLR/CRP/PLR for OS. The Begg's *p* and Egger's *p* for OS were 0.115 and 0.008, respectively. Calculate new HR using trim and fill methods (HR: 1.80; 95% CI: 1.42–2.28; *p* < 0.001; random effects). No publication bias was observed in the prognostic value of CRP for OS. The Begg's *p* and Egger's *p* for OS were 1.000 and 0.748. Among the seven included studies for PLR on OS, the Egger's test depicted proof of publication bias (*p* = 0.000), whereas the Begg's test did not (*p* = 0.144). Therefore, we used the trim and fill method allowing the new HRs to retain statistical significance (HR: 1.58; 95% CI: 1.17–2.13; *p* < 0.001; random effects).

## Conclusions

Our research shows that hematological markers are one of the important prognostic indicators for patients affected by high-grade STS and patients with the STS being located in the extremity. Large-scale prospective studies are needed, especially studies targeting specific STS subtypes, to further validate our results.

## Author Contributions

L-QL collected and analyzed the data and wrote the paper. Z-HB and L-HZ assisted in collecting the data and participated in the writing. YaZ, X-CL, YiZ, JW, and Y-KL assisted in the design of this study. J-ZL was responsible for all the integrity of data and the accuracy of data analysis. All authors have thoroughly revised the manuscript.

### Conflict of Interest

The authors declare that the research was conducted in the absence of any commercial or financial relationships that could be construed as a potential conflict of interest.

## References

[1] Siegel RL Miller KD Jemal A Cancer statistics, 2019. CA Cancer J Clin. (2019) 69:7–34. 10.3322/caac.21551 30620402

[2] Cormier JN Pollock RE. Soft tissue sarcomas. CA Cancer J Clin. (2004) 54:94–109. 10.3322/canjclin.54.2.94 15061599

[3] Stojadinovic A Leung DH Hoos A Jaques DP Lewis JJ Brennan MF. Analysis of the prognostic significance of microscopic margins in 2,084 localized primary adult soft tissue sarcomas. Ann Surg. (2002) 235:424–34. 10.1097/00000658-200203000-00015 11882765 PMC1422449

[4] Kattan MW Leung DH Brennan MF. Postoperative nomogram for 12-year sarcoma-specific death. J Clin Oncol. (2002) 20:791–6. 10.1200/JCO.20.3.791 11821462

[5] Mantovani A Allavena P Sica A Balkwill F. Cancer-related inflammation. Nature. (2008) 454:436–44. 10.1038/nature07205 18650914

[6] Grivennikov SI Greten FR Karin M. Immunity, inflammation, and cancer. Cell. (2010) 140:883–99. 10.1016/j.cell.2010.01.025 20303878 PMC2866629

[7] Hefler LA Concin N Hofstetter G Marth C Mustea A Sehouli J. Serum C-reactive protein as independent prognostic variable in patients with ovarian cancer. Clin Cancer Res. (2008) 14:710–4. 10.1158/1078-0432.CCR-07-1044 18245530

[8] Templeton AJ McNamara MG Šeruga B Vera-Badillo FE Aneja P Ocaña A. Prognostic role of neutrophil-to-lymphocyte ratio in solid tumors: a systematic review and meta-analysis. J Natl Cancer Inst. (2014) 106:dju124. 10.1093/jnci/dju124 24875653

[9] Polterauer S Grimm C Tempfer C Sliutz G Speiser P Reinthaller A. C-reactive protein is a prognostic parameter in patients with cervical cancer. Gynecol Oncol. (2007) 107:114–7. 10.1016/j.ygyno.2007.06.001 17617445

[10] Zhou L Cai X Liu Q Jian ZY Li H Wang KJ. Prognostic role of C-reactive protein in urological cancers: a meta-analysis. Sci Rep. (2015) 5:12733. 10.1038/srep12733 26235332 PMC4522672

[11] Nishijima TF Muss HB Shachar SS Tamura K Takamatsu Y. Prognostic value of lymphocyte-to-monocyte ratio in patients with solid tumors: a systematic review and meta-analysis. Cancer Treat Rev. (2015) 41:971–8. 10.1016/j.ctrv.2015.10.003 26481060

[12] McMillan DC. The systemic inflammation-based Glasgow Prognostic Score: a decade of experience in patients with cancer. Cancer Treat Rev. (2013) 39:534–40. 10.1016/j.ctrv.2012.08.003 22995477

[13] Wang X Ni X Tang G. Prognostic role of platelet-to-lymphocyte ratio in patients with bladder cancer: a meta-analysis. Front Oncol. (2019) 9:757. 10.3389/fonc.2019.00757 31475109 PMC6703229

[14] Stang A Critical evaluation of the Newcastle-Ottawa scale for the assessment of the quality of non-randomized studies in meta-analyses. Eur J Epidemiol. (2010) 25:603–5. 10.1007/s10654-010-9491-z 20652370

[15] DerSimonian R Laird N. Meta-analysis in clinical trials. Control Clin Trials. (1986) 7:177–88. 10.1016/0197-2456(86)90046-2 3802833

[16] Mantel N Haenszel W. Statistical aspects of the analysis of data from retrospective studies of disease. J Natl Cancer Inst. (1959) 22:719–48. 13655060

[17] Begg CB Mazumdar M. Operating characteristics of a rank correlation test for publication bias. Biometrics. (1994) 50:1088–101. 10.2307/2533446 7786990

[18] Idowu OK Ding Q Taktak AF Chandrasekar CR Yin Q. Clinical implication of pretreatment neutrophil to lymphocyte ratio in soft tissue sarcoma. Biomarkers. (2012) 17:539–44. 10.3109/1354750X.2012.699554 22793493

[19] Marshall S Nakano K Sugiura Y Taira S Ono M Tomomatsu J. Outcome for advanced or metastatic soft tissue sarcoma of nonextremities treated with doxorubicin-based chemotherapy: a retrospective study from a single cancer institution. Sarcoma. (2018) 2018:8926598. 10.1155/2018/8926598 29849480 PMC5932429

[20] Nakamura T Grimer R Gaston C Francis M Charman J Graunt P. The value of C-reactive protein and comorbidity in predicting survival of patients with high grade soft tissue sarcoma. Eur J Cancer. (2013) 49:377–85. 10.1016/j.ejca.2012.09.004 23058786

[21] Szkandera J Gerger A Liegl-Atzwanger B Absenger G Stotz M Friesenbichler J. The lymphocyte/monocyte ratio predicts poor clinical outcome and improves the predictive accuracy in patients with soft tissue sarcomas. Int J Cancer. (2014) 135:362–70. 10.1002/ijc.28677 24347236

[22] Panotopoulos J Posch F Alici B Funovics P Stihsen C Amann G. Hemoglobin, alkalic phosphatase, and C-reactive protein predict the outcome in patients with liposarcoma. J Orthop Res. (2015) 33:765–70. 10.1002/jor.22827 25641201

[23] Jiang L Jiang S Situ D Lin Y Yang H Li Y. Prognostic value of monocyte and neutrophils to lymphocytes ratio in patients with metastatic soft tissue sarcoma. Oncotarget. (2015). 6:9542–50. 10.18632/oncotarget.3283 25865224 PMC4496237

[24] Nakamura T Katagiri H Shido Y Hamada S Yamada K Nagano A. Analysis of factors for predicting survival in soft-tissue sarcoma with metastatic disease at initial presentation. Anticancer Res. (2017) 37:3137–41. 10.21873/anticanres.11671 28551655

[25] Chan JY Zhang Z Chew W Tan GF Lim CL Zhou L. Biological significance and prognostic relevance of peripheral blood neutrophil-to-lymphocyte ratio in soft tissue sarcoma. Sci Rep. (2018) 8:11959. 10.1038/s41598-018-30442-5 30097600 PMC6086886

[26] Park G Song SY Ahn JH Kim WL Lee JS Jeong SY. The pretreatment erythrocyte sedimentation rate predicts survival outcomes after surgery and adjuvant radiotherapy for extremity soft tissue sarcoma. Radiat Oncol. (2019) 14:116. 10.1186/s13014-019-1331-z 31272506 PMC6610892

[27] Sasaki H Nagano S Komiya S Taniguchi N Setoguchi T. Validation of different nutritional assessment tools in predicting prognosis of patients with soft tissue spindle-cell sarcomas. Nutrients. (2018) 10:e765. 10.3390/nu10060765 29899304 PMC6024570

[28] Liang Y Xiao W Guan YX Wang W Chen HY Fang C. Prognostic value of the C-reactive protein/Albumin Ratio (CAR) in patients with operable soft tissue sarcoma. Oncotarget. (2017) 8:98135–47. 10.18632/oncotarget.20990 29228679 PMC5716719

[29] Maretty-Kongstad K Aggerholm-Pedersen N Keller J Safwat A A validated prognostic biomarker score for adult patients with non-metastatic soft tissue sarcomas of the trunk and extremities. Transl Oncol. (2017) 10:942–8. 10.1016/j.tranon.2017.09.002 29031130 PMC5643061

[30] Nakamura T Matsumine A Asanuma K Matsubara T Sudo A. The value of the high-sensitivity modified Glasgow prognostic score in predicting the survival of patients with a soft-tissue sarcoma. Bone Joint J. (2015) 97-B:847–52. 10.1302/0301-620X.97B.35098 26033068

[31] Szkandera J Gerger A Liegl-Atzwanger B Absenger G Stotz M Samonigg H. Validation of the prognostic relevance of plasma C-reactive protein levels in soft-tissue sarcoma patients. Br J Cancer. (2013) 109:2316–22. 10.1038/bjc.2013.595 24084772 PMC3817333

[32] Choi ES Kim HS Han I. Elevated preoperative systemic inflammatory markers predict poor outcome in localized soft tissue sarcoma. Ann Surg Oncol. (2014) 21:778–85. 10.1245/s10434-013-3418-3 24306668

[33] García-Ortega DY Álvarez-Cano A Sánchez-Llamas LA Caro-Sanchez C Martínez-Said H Luna-Ortiz K. Neutrophil/lymphocyte ratio is associated with survival in synovial sarcoma. Surg Oncol. (2018) 27:551–5. 10.1016/j.suronc.2018.07.012 30217318

[34] Chen S Luo P Yang L Zheng B Sun Z Yan W. Prognostic analysis of surgically treated clear cell sarcoma: an analysis of a rare tumor from a single center. Int J Clin Oncol. (2019) 24:1605–11. 10.1007/s10147-019-01487-x 31243628 PMC6861539

[35] Willegger M Posch F Schieder S Funovics PT Scharrer A Brodowicz T. Serum creatinine and albumin predict sarcoma-specific survival in patients with myofibroblastic and fibroblastic sarcomas. J Orthop Res. (2017) 35:2815–24. 10.1002/jor.23598 28485477

[36] Tsuda Y Ogura K Kobayashi E Hiruma T Iwata S Asano N. Impact of geriatric factors on surgical and prognostic outcomes in elderly patients with soft-tissue sarcoma. Jpn J Clin Oncol. (2017) 47:422–9. 10.1093/jjco/hyx016 28201801

[37] Vasquez L León E Beltran B Maza I Oscanoa M Geronimo J. Pretreatment neutrophil-to-lymphocyte ratio and lymphocyte recovery: independent prognostic factors for survival in pediatric sarcomas. J Pediatr Hematol Oncol. (2017) 39:538–46. 10.1097/MPH.0000000000000911 28697168

[38] Nakamura T Matsumine A Matsubara T Asanuma K Yada Y Hagi T. Infiltrative tumor growth patterns on magnetic resonance imaging associated with systemic inflammation and oncological outcome in patients with high-grade soft-tissue sarcoma. PLoS ONE. (2017) 12:e0181787. 10.1371/journal.pone.0181787 28727824 PMC5519204

[39] Nakamura T Matsumine A Matsubara T Asanuma K Uchida A Sudo A. Clinical significance of pretreatment serum C-reactive protein level in soft tissue sarcoma. Cancer. (2012) 118:1055–61. 10.1002/cncr.26353 21761398

[40] Cheng Y Mo F Pu L Li Q Ma X. Pretreatment inflammatory indexes as prognostic predictors of survival in patients suffering from synovial sarcoma. Front Oncol. (2019) 9:955. 10.3389/fonc.2019.00955 31608240 PMC6769112

[41] Denkert C Loibl S Noske A Roller M Müller BM Komor M. Tumor-associated lymphocytes as an independent predictor of response to neoadjuvant chemotherapy in breast cancer. J Clin Oncol. (2010) 28:105–13. 10.1200/JCO.2009.23.7370 19917869

[42] De Vita A Mercatali L Recine F Pieri F Riva N Bongiovanni A. Current classification, treatment options, and new perspectives in the management of adipocytic sarcomas. Onco Targets Ther. (2016) 9:6233–46. 10.2147/OTT.S112580 27785071 PMC5067014

[43] Gluck WL Gounder MM Frank R Eskens F Blay JY Cassier PA Phase 1 study of the MDM2 inhibitor AMG 232 in patients with advanced P53 wild-type solid tumors or multiple myeloma. Invest New Drugs. (2019). 10.1007/s10637-019-00840-1. [Epub ahead of print]. PMC7211202 31359240

[44] Lahat G Tuvin D Wei C Wang WL Pollock RE Anaya DA. Molecular prognosticators of complex karyotype soft tissue sarcoma outcome: a tissue microarray-based study. Ann Oncol. (2010) 21:1112–20. 10.1093/annonc/mdp459 19875755

[45] Kandel RA Yao X Dickson BC Ghert M Popovic S Purgina BM. Molecular analyses in the diagnosis and prediction of prognosis in non-GIST soft tissue sarcomas: a systematic review and meta-analysis. Cancer Treat Rev. (2018) 66:74–81. 10.1016/j.ctrv.2018.04.005 29709714

[46] Salah S Lewin J Amir E Abdul Razak A. Tumor necrosis and clinical outcomes following neoadjuvant therapy in soft tissue sarcoma: a systematic review and meta-analysis. Cancer Treat Rev. (2018) 69:1–10. 10.1016/j.ctrv.2018.05.007 29843049

[47] Kubo T Furuta T Johan MP Ochi M. Prognostic significance of (18)F-FDG PET at diagnosis in patients with soft tissue sarcoma and bone sarcoma; systematic review and meta-analysis. Eur J Cancer. (2016) 58:104–11. 10.1016/j.ejca.2016.02.007 26990930

[48] Coussens LM Werb Z. Inflammation and cancer. Nature. (2002) 420:860–7. 10.1038/nature01322 12490959 PMC2803035

[49] Balkwill F Mantovani A. Inflammation and cancer: back to Virchow. Lancet. (2001) 357:539–45. 10.1016/S0140-6736(00)04046-0 11229684

[50] De Larco JE Wuertz BR Furcht LT. The potential role of neutrophils in promoting the metastatic phenotype of tumors releasing interleukin-8. Clin Cancer Res. (2004) 10:4895–900. 10.1158/1078-0432.CCR-03-0760 15297389

[51] Kitamura T Qian BZ Pollard JW. Immune cell promotion of metastasis. Nat Rev Immunol. (2015) 15:73–86. 10.1038/nri3789 25614318 PMC4470277

[52] Elinav E Nowarski R Thaiss CA Hu B Jin C Flavell RA. Inflammation-induced cancer: crosstalk between tumours, immune cells and microorganisms. Nat Rev Cancer. (2013) 13:759–71. 10.1038/nrc3611 24154716

[53] Liu G Ke LC Sun SR. Prognostic value of pretreatment neutrophil-to-lymphocyte ratio in patients with soft tissue sarcoma: a meta-analysis. Medicine. (2018) 97:e12176. 10.1097/MD.0000000000012176 30200120 PMC6133428

[54] Li Y Liu X Zhang J Yao W. Prognostic role of elevated preoperative systemic inflammatory markers in localized soft tissue sarcoma. Cancer Biomark. (2016) 16:333–42. 10.3233/CBM-160571 26835589 PMC13016492

[55] Wang X Liu S Zhao X Fang E Zhao X. The value of C-reactive protein as an independent prognostic indicator for disease-specific survival in patients with soft tissue sarcoma: a meta-analysis. PLoS ONE. (2019) 14:e0219215. 10.1371/journal.pone.0219215 31260491 PMC6602474

[56] Goubran HA Burnouf T Radosevic M El-Ekiaby M. The platelet-cancer loop. Eur J Intern Med. (2013) 24:393–400. 10.1016/j.ejim.2013.01.017 23433737

[57] Gay LJ Felding-Habermann B. Contribution of platelets to tumour metastasis. Nat Rev Cancer. (2011) 11:123–34. 10.1038/nrc3004 21258396 PMC6894505

[58] Sullivan LA Brekken RA. The VEGF family in cancer and antibody-based strategies for their inhibition. MAbs. (2010) 2:165–75. 10.4161/mabs.2.2.11360 20190566 PMC2840235

[59] Bingle L Brown NJ Lewis CE. The role of tumour-associated macrophages in tumour progression: implications for new anticancer therapies. J Pathol. (2002) 196:254–65. 10.1002/path.1027 11857487

